# Association between Accessory Gene Regulator Polymorphism and Mortality among Critically Ill Patients Receiving Vancomycin for Nosocomial MRSA Bacteremia: A Cohort Study

**DOI:** 10.1155/2016/8163456

**Published:** 2016-05-15

**Authors:** Angélica Cechinel, Denise P. Machado, Eduardo Turra, Dariane Pereira, Rodrigo P. dos Santos, Regis G. Rosa, Luciano Z. Goldani

**Affiliations:** ^1^Infectious Diseases Unit, Hospital de Clínicas de Porto Alegre, Universidade Federal do Rio Grande do Sul, 90035-903 Porto Alegre, RS, Brazil; ^2^Hospital Infection Control Section, Hospital de Clínicas de Porto Alegre, Universidade Federal do Rio Grande do Sul, 90035-903 Porto Alegre, RS, Brazil

## Abstract

*Background*. Polymorphism of the accessory gene regulator group II (*agr*) in methicillin-resistant* Staphylococcus aureus* (MRSA) is predictive of vancomycin failure therapy. Nevertheless, the impact of group II* agr* expression on mortality of patients with severe MRSA infections is not well established.* Objective*. The goal of our study was to evaluate the association between* agr* polymorphism and all-cause in-hospital mortality among critically ill patients receiving vancomycin for nosocomial MRSA bacteremia.* Methods*. All patients with documented bacteremia by MRSA requiring treatment in the ICU between May 2009 and November 2011 were included in the study. Cox proportional hazards regression was performed to evaluate whether* agr* polymorphism was associated with all-cause in-hospital mortality. Covariates included age, APACHE II score, initial C-reactive protein plasma levels, initial serum creatinine levels, vancomycin minimum inhibitory concentration, vancomycin serum levels, and time to effective antibiotic administration.* Results*. The prevalence of group I and group II* agr* expression was 52.4% and 47.6%, respectively. Bacteremia by MRSA group III or group IV* agr* was not documented in our patients. The mean APACHE II of the study population was 24.3 (standard deviation 8.5). The overall cohort mortality was 66.6% (14 patients). After multivariate analysis, initial plasma C-reactive protein levels (*P* = 0.01), initial serum creatinine levels (*P* = 0.008), and expression of group II* agr* (*P* = 0.006) were positively associated with all-cause in-hospital mortality. Patients with bacteremia by MRSA with group II* agr* expression had their risk of death increased by 12.6 times when compared with those with bacteremia by MRSA with group I* agr* expression.* Conclusion*. Group II* agr* polymorphism is associated with an increase in mortality in critically ill patients with bacteremia by MRSA treated with vancomycin.

## 1. Introduction

Methicillin-resistant* Staphylococcus aureus* (MRSA) is well recognized as a major cause of nosocomial infection worldwide. Its strong adaptive power to antibiotics has resulted in the emergence of methicillin-resistant* S*.* aureus* (MRSA) [1, 2]. Resistance to methicillin and other *β*-lactam antibiotics is caused by the mecA gene, which is situated on a mobile genetic element, the staphylococcal cassette chromosome mec (SCCmec) [3]. Although the origin of MRSA is not fully understood, it is suspected that methicillin-susceptible* S*.* aureus* (MSSA) acquired the* mecA* gene through horizontal transfer from coagulase-negative staphylococci. Recent studies have shown that overall in-hospital mortality rates for patients with bloodstream infections due to MRSA are in the range of 30% but can be as high as 65% in some centers [4, 5]. A thorough knowledge of the epidemiology and the molecular epidemiology of MRSA strains is required to develop effective strategies to prevent the spread of MRSA.

The aim of the present study was to evaluate the association between* agr* polymorphism and all-cause in-hospital mortality among critically ill patients receiving vancomycin for nosocomial MRSA bacteremia.

## 2. Methods

A retrospective cohort was performed at a 30-bed general intensive care unit (ICU) of an 845-bed, university-affiliated tertiary care hospital located in the southernmost state of Brazil. The medical records of all cases of documented bacteremia by MRSA requiring vancomycin treatment in the ICU between May 2009 and November 2011 were evaluated. MRSA bacteremia was defined as the presence of at least one positive blood culture for MRSA in a blood sample from a patient with clinical findings consistent with infection [6]. Blood cultures were performed by inoculating 5–10 mL of blood into a flask of the automatic commercial system Bactec/Alert® (Vitek system). Positive cultures were further subcultured in Mueller-Hinton agar (Isofar Ltd., Brazil) supplemented with 5% of human blood and incubated for 24–48 h at 35 ± 2°C in the hospital microbiology laboratory. Initial susceptibility testing for oxacillin resistance was performed according to CLSI guidelines, using a 30 *μ*g cefoxitin disc in Mueller-Hinton agar [7]. Individual isolates were stored in trypticase soy broth with 20% glycerol at −80°C until MIC testing was performed. MICs for vancomycin, daptomycin, linezolid, quinupristin-dalfopristin, and tigecycline were determined by the Etest (bioMérieux), according to the manufacturer's guidelines (AB Biodisk). Daptomycin, quinupristin-dalfopristin, and linezolid resistance were defined as an isolate with an MIC greater than 1 mcg/mL, 1 mcg/mL, and 4 mcg/mL, respectively [7, 8]. There are currently no recommended CLSI (Clinical and Laboratory Standards Institute) interpretative criteria for tigecycline. In addition, the MICs of vancomycin were determined in duplicate by reference broth microdilution method, as recommended by CLSI, using in-house prepared panels. The following dilutions of vancomycin were tested: 16, 8, 4, 2, 1, 0.5, 0.25, and 0.125 mcg/mL. According to current CLSI criteria, vancomycin-intermediate* S. aureus* and vancomycin-resistant* S*.* aureus* are currently defined using BMD as exhibiting vancomycin MICs of 4 to 8 mcg/mL and ≥16 mcg/mL, respectively. The Etest procedure was performed using a suspension of each isolate in Mueller-Hinton broth, adjusted to the density of a 0.5 McFarland standard, and was swabbed in three directions to ensure uniform growth onto Mueller-Hinton agar plates. The MIC was read where inhibition of growth intersected the Etest strip. When small colonies grew within the zone of inhibition or a haze of growth occurred around MIC endpoints, the highest MIC intersection was recorded. MRSA isolates were characterized by molecular typing techniques. The typing of staphylococcal cassette chromosome mec (SCCmec) was performed using the multiplex PCR method described by Boye et al.; four sets of primers were used for amplification of the target DNA [9]. PCR was also used to characterize accessory gene regulator (agr) based on the method described by Gilot et al., who developed a method for typing of agr group by a PCR reaction involving a primer common to all groups and four primers, specific for each group agr (agr I–IV) [10].

### 2.1. Statistical Analysis

The results were described as frequency (%) in the case of discrete variables. Clinical and microbiological characteristics and therapeutic interventions were included in the univariate and multivariate analyses. Cox proportional hazards regression was performed to evaluate whether* agr* polymorphism (determined by PCR) was associated with all-cause 30-day hospital mortality. Covariates included age, APACHE II score (acute physiological assessment and chronic health evaluation), initial C-reactive protein plasma levels, initial serum creatinine levels, vancomycin minimum inhibitory concentration, and time to effective antibiotic administration. All variables with a *P* value < 0.10 in the univariate analysis were included in the stepwise multiple logistic regression to identify potential factors associated with mortality. In the multivariate model, independent variables were eliminated from the highest to the lowest *P* value but remained in the model if the *P* value was <0.05.

The study was approved by the Ethics and Research Committee of Hospital de Clínicas de Porto Alegre, Brazil. Because no direct patient contact was planned, the requirement for informed consent was waived.

## 3. Results

**Figure 1:**
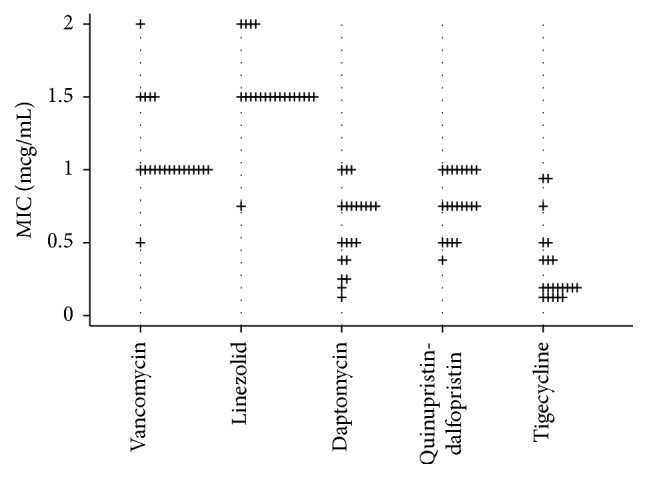
Distribution of specific antibiotic minimum inhibitory concentrations (MICs) by Etest for MRSA blood isolates.

**Figure 2:**
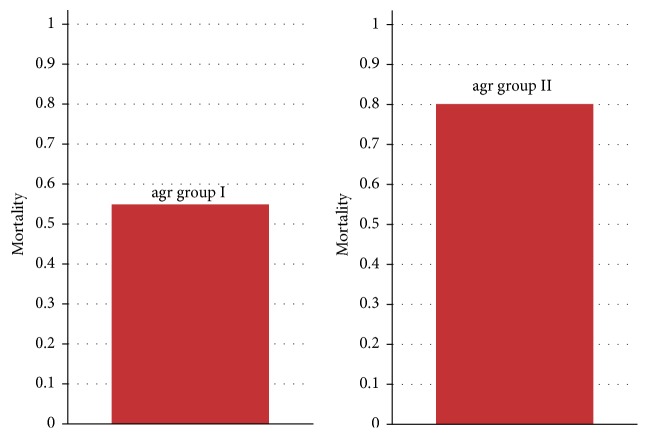
In-hospital mortality of critically ill patients with MRSA bacteremia treated with vancomycin according accessory gene regulator (*agr*) polymorphism. Values expressed as deaths/survival cases.

In total, 21 patients with MRSA bacteremia were evaluated during the study period. The baseline characteristics are shown in Table 1. The prevalence of group I and group II* agr* expression was 52.4% and 47.6%, respectively. No case of bacteremia by MRSA group III or group IV* agr* was documented. The mean APACHE II of the study population was 24.3 (standard deviation 8.5). The overall cohort mortality was 66.6% (14 patients). Distribution of specific antibiotic minimum inhibitory concentrations (MICs) to vancomycin, linezolid, daptomycin, quinupristin-dalfopristin, and tigecycline for MRSA blood isolates is presented in Figure 1. MRSA isolates were overall susceptible for all the antimicrobials. The majority of the MRSA isolates presented vancomycin MICs by Etest testing between 1.0 and 1.5 mcg/mL. The MICs for vancomycin determined using the broth microdilution method were ≤1.0 mcg/mL. Univariate analysis of possible relevant factors in patients associated with mortality in patients with MRSA bacteremia is shown in Table 2. After multivariate analysis, initial plasma C-reactive protein levels (*P* = 0.10), initial serum creatinine levels (*P* = 0.008), and expression of group II* agr* (*P* = 0.006) were positively associated with all-cause in-hospital mortality (Table 3). Patients with bacteremia by MRSA with group II* agr* expression had their risk of death increased by 12.6 times when compared with those with bacteremia by MRSA with group I* agr* expression. In-hospital mortality of critically ill patients with MRSA bacteremia treated with vancomycin according accessory gene regulator (*agr*) polymorphism is shown in Figure 2.

## 4. Discussion

MRSA comprised nearly 60% of* S*.* aureus* organisms isolated in US intensive care units (ICUs) in 2003 [11]. In Latin America, rates of MRSA surpassed 50% in over half of the countries, and a similar situation was observed in many institutions from the Asia-Pacific region [12]. In Brazil, the Antimicrobial Surveillance Program (SENTRY) described a prevalence of MRSA bacteraemia of 30.9% in hospitalized patients between 1997 and 2000, but in large Brazilian teaching hospitals, up to 73% of clinically significant* S*.* aureus* bacteremia was caused by methicillin-resistant strains [13, 14].

Our patients were infected with MRSA isolates presenting low MICs against vancomycin. Therefore, we could not find any association between 30-day mortality and MRSA vancomycin MICs. Even considering the important comorbidities and high APACHE II scores typically present in ICU patients, the overall mortality of our patients with MRSA bacteremia treated with vancomycin was considered high. Recently, infections due to isolates with high but susceptible vancomycin MICs have been associated with additional treatment failures and patient mortality [15].

On the other hand, a recent meta-analysis has shown that there were no statistically significant differences in the risk of death when comparing patients with* S. aureus* exhibiting high-vancomycin MIC to those with low-vancomycin MIC (<1.5 mcg/mL) [16]. These poorer outcomes may in part be explained by severity of comorbidities and inability of attaining appropriate vancomycin levels in these patients. However, assumptions that these poor outcomes are solely due to failure to achieve optimal serum levels of vancomycin are premature. The availability of effective alternatives further erodes the position of vancomycin as first-line therapy. The emergence of resistance and cost considerations, however, favor a more measured approach when using alternative antimicrobials. However, our MRSA isolates did not present MICs > 2 mcg/mL and the serum levels of vancomycin attained by our patients were within the optimal range of 15.0–20.0 *μ*g/mL (data not shown). In addition, all MRSA strains isolated from the blood of our patients were susceptible to the available alternative antimicrobials. Although uncommon, it is important to mention that MRSA resistance to new antibiotics such as linezolid and daptomycin has been described in clinical settings [17, 18].

In our study, agr II MRSA was associated with increased mortality in patients with bacteremia. The accessory gene regulator (Agr) is a quorum-sensing regulator in* S. aureus* that is responsible for biofilm production and the expression of adherence and virulence factors. Previous studies have found that MRSA isolates with an agr II polymorphism may influence the clinical efficacy of vancomycin [19, 20]. SCCmec type II has been shown to be a marker for disease severity and mortality [21]. Most of our MRSA isolates were nontypeable (53%). Eleven isolates (38%) carried SCCmec element types III and IV. Because of the small sample sizes, we could not evaluate in our multivariable logistic regression model the association of SCCmec type with hospital mortality.

Several points must be considered when analyzing our data. We were not able to control for other mortality risk factors in our sample. In addition, the observations in this study are subject to limitations due to the fact that the data was obtained from a retrospective analysis.

In conclusion, expression of group II* agr* poses risk for mortality in critically ill patients with bacteremia by MRSA treated with vancomycin. Alternative antimicrobial agents including daptomycin and linezolid for treatment of MRSA bacteremia expressing group II* agr* should be considered in this setting.

## Figures and Tables

**Table 1:** Characteristics of the patients with MRSA bacteremia.

Variable	*N* (%)
Age years, mean (SD)	55.5 (17.2)
Female sex, number (%)	9 (45.0)
Type of underlying disease, number (%)	
Diabetes mellitus	6 (28.6)
Malignancy	5 (23.8)
Cardiac failure	3 (14.3)
Chronic renal failure	3 (14.3)
Chronic obstructive pulmonary disease	2 (9.5)
HIV	2 (9.5)
APACHE II score, mean (SD)	24.3 (8.5)
Initial plasma CRP, mg/L, mean (SD)	178.6 (110.8)
Initial serum creatinine, g/dL, mean (SD)	1.7 (1.3)
Genotypic characteristic of blood isolates, number (%)	
*agr* group	
I	11 (52.4)
II	10 (47.6)
III	0 (0)
IV	0 (0)
SCC*mec* type	
I	2 (9.5)
II	0 (0)
III	5 (23.8)
IV	3 (14.3)
ND	11 (52.4)
Overall in-hospital mortality, number (%)	14 (66.6)

*Note.* SD: standard deviation; CRP: C-reactive protein; *agr*: accessory gene regulator; SCC*mec*: staphylococcal cassette chromosome *mec*; ND: not determined.

**Table 2:** Univariate Cox regression analysis of risk factors for in-hospital mortality in critically ill patients with MRSA bacteremia treated with vancomycin.

Variable	Mortality group (*n* = 14)	Survival group (*n* = 7)	HR (95% CI)	*P* value
Age years, median (SD)	57.1 (13.6)	52.4 (23.8)	1.01 (0.97–1.05)	0.47
APACHE II score, median (SD)	25.0 (9.1)	23.1 (7.8)	1.01 (0.93–1.11)	0.69
Initial plasma CRP, mg/L, median (SD)	226.0 (100.5)	90.6 (69.2)	1.004 (0.99–1.009)	0.07
Initial serum creatinine, g/dL, mean (SD)	1.9 (1.2)	1.2 (1.4)	1.42 (0.99–2.04)	0.05
MIC for vancomycin > 1 mcg/mL, No (%)	1 (7.1)	4 (57.1)	0.23 (0.03–1.85)	0.17
Appropriate vancomycin serum trough levels, number (%)	8 (57.1)	2 (14.2)	3.05 (0.37–25.11)	0.29
Time to vancomycin administration, days, mean (SD)	0.38 (0.96)	2.33 (2.33)	0.66 (0.37–1.16)	0.15
Group II *agr* specificity, number (%)	8 (57.1)	2 (28.5)	2.80 (0.84–9.38)	0.09

*Note.* HR: hazard ratio; 95% CI: 95% confidence interval; SD: standard deviation; CRP: C-reactive protein; MIC: minimum inhibitory concentration; *agr*: accessory gene regulator.

**Table 3:** Multivariate Cox regression analysis of factors associated with in-hospital mortality in critically ill patients with MRSA bacteremia treated with vancomycin.

Variable	Adjusted HR	95% CI	*P* value
Initial plasma CRP, mg/L	1.01	1.002–1.019	0.01
Initial serum creatinine, g/dL	2.11	1.21–3.68	0.008
Group II *agr* specificity	12.60	2.07–76.69	0.006

*Note.* HR: hazard ratio; 95% CI: 95% confidence interval; CRP: C-reactive protein; *agr*: accessory gene regulator.
